# Seprafilm^®^ adhesion barrier: (2) a review of the clinical literature on intraabdominal use

**DOI:** 10.1007/s10397-012-0742-8

**Published:** 2012-04-15

**Authors:** Michael P. Diamond, Ellen L. Burns, Beverly Accomando, Sadiqa Mian, Lena Holmdahl

**Affiliations:** 1 Department of Obstetrics and Gynecology, Division of Reproductive Endocrinology and Infertility, Wayne State University, 60 West Hancock, Detroit, MI 48201 USA; 2 Genzyme Corporation, Cambridge, MA USA

**Keywords:** Postoperative adhesions, Seprafilm, Anti-adhesion adjuvant, Adhesiolysis

## Abstract

This study seeks to provide a review of the clinical data published as of July 2011 concerning the postsurgical adhesion barrier, Seprafilm (chemically modified hyaluronic acid and carboxymethylcelulose; Genzyme Corporation, Cambridge, MA). Included articles detail the application of Seprafilm for intraabdominal uses that have been approved (on-label) and those considered investigational (off-label) by the FDA. Medline and EMBASE Drugs and Pharmaceuticals databases were searched for all original clinical Seprafilm research published as of July 2011. All human Seprafilm intraabdominal clinical reports and studies, excluding those related to prosthetic mesh were included. Data extraction involved the systematic review of each article. The data synthesis is the summary of Seprafilm human intraabdominal clinical reports and studies describing safety and/or efficacy. The safety and efficacy of Seprafilm in reducing postoperative adhesions has been clearly demonstrated in abdominal and pelvic laparotomy. While reports have described the safe and successful use of Seprafilm following laparoscopy, pediatric laparotomy, and in patients with malignancy and/or infection, the safety and efficacy of Seprafilm use in these procedures has not been definitively established in randomized controlled trials.

## Introduction

Seprafilm adhesion barrier is a bioresorbable membrane composed of chemically modified sodium hyaluronate (HA) and carboxymethylcellulose (CMC). The absorbable adhesion barrier received FDA market approval in 1996 and is available worldwide. To date, over two million patients have been treated with Seprafilm. The product is indicated for use in adult patients undergoing abdominal or pelvic laparotomy and is intended to reduce the incidence, extent, and severity of postoperative adhesions between the abdominal wall and omentum, small bowel, bladder, and stomach; and between the uterus and tubes, ovaries, large bowel, and bladder [1]. Postsurgical adhesions are also known to be a source of complications following abdominal and pelvic laparoscopy [2], pediatric procedures as well as in surgical patients with infections and malignancy. Consequently, Seprafilm has been used and studied “off-label,” at the discretion of individual surgeons, following many of these types of surgeries.

In an attempt to improve upon the outcomes achievable through the use of good surgical technique, anti-adhesion adjuvants have been introduced. This article intends to summarize the available research, as of July 2011, for one such adjuvant: Seprafilm Adhesion Barrier (Genzyme, Cambridge, MA) following abdominal and pelvic procedures considered both “on” and “off-label.”

## Clinical safety and efficacy studies

For over 15 years, Seprafilm’s effect on adhesion development has been evaluated in men, women, and children by multiple clinical investigators. The reported results of these studies have largely shown, often at repeat surgery, that Seprafilm is effective in reducing adhesion development or has led to another beneficial outcome. An overview of these published clinical reports is provided in Table 1.

**Table 1:** Clinical publications and calculated effect size

Reference	*N*	Therapeutic area	Favorable seprafilm outcome	Effect size^a^	Reported *p* value
Becker et al. [3]	183	Abdominal	Incidence	17.4	<0.00000000001
			Incidence of fewer dense adhesions	7.6	<0.0001
			Incidence of fewer moderate adhesions	16.5	
			Incidence of greater mild adhesions	8.5	
			Extent	1.2	<0.001
Fukushima et al. [5]	30	Abdominal	Comparison to untreated control site		
			Incidence of adhesions	3.0	0.05
			Incidence of more grade 1	3.3	0.05
			Incidence of fewer grade 3	2.1	
			Comparison to pts with previous laparotomy		
			Incidence of adhesions	3.6	0.05
			Incidence of more grade 1	9.0	0.01
			Incidence of fewer grade 3	3.5	
Salum et al. [14]	538	Abdominal	Incidence of intestinal obstruction	1.5	NS
			Incidence of enterolysis	2.6	NS
			Successful conservative management of bowel obstruction	1.1	NS
Vriland et al. [30]	71	Abdominal	Incidence of adhesions entire incision	Cannot be calculated—0 events in control group	0.48
			Incidence of pelvic adhesions	3.0	0.41
			Incidence of adhesions superior segment	2.1	0.48
			Incidence of adhesions middle segment	8.0	0.09
			Incidence of adhesions inferior segment	3.0	0.28
			Median severity score entire incision	Cannot be calculated—only range reported	0.002
Tang et al. [27]	181	Abdominal	Overall mean 4 quadrant adhesion score Phase I	−0.06	NS
			Overall mean 4 quadrant adhesion score Phase II	0.8	0.02
Kudo et al. [15]	51	Abdominal	Incidence of early postoperative bowel obstruction	Cannot be calculated—0 events in Seprafilm group	<0.05
			Resumed liquid diet sooner	0.6	NS
			Resumed solid diet sooner	0.5	NS
Mohri et al. [16]	367	Abdominal	Incidence of early postoperative small bowel obstruction	2.4	0.02
			Reoperation for early postoperative small bowel obstruction	2.7	NS
Fazio et al. [8]	1791	Abdominal	Incidence of operative adhesive small bowel obstruction	1.9	0.044
			Incidence of all cause bowel obstruction	1.0	NS
Salum et al. [28]	191	Abdominal	Incidence of grade 0 adhesions midline and stoma	4.3	0.021
			Incidence of grade 0 adhesions midline	3.1	
			Incidence of greater grade 1 adhesions midline and stoma	2.0	0.096
			Incidence of greater grade 1 adhesions midline	1.1	
			Incidence of fewer grade 3 adhesions midline and stoma	2.56	
			Incidence of fewer grade 3 adhesions midline	1.2	
			Incidence of enterotomy/myotomy midline and stoma	0.7	
			Incidence of enterotomy/myotomy midline	0.8	
Fujii et al. [9]	115	Abdominal	Incidence of adhesion related post-op ileus	1.0	NS
	27		Incidence of adhesions	11.6	0.0004
Van der Wal et al. [31]	35	Abdominal	Incidence of chronic abdominal complaints	4.7	0.018
			Incidence of small bowel obstruction	Cannot be calculated—0 events in treatment group	NS
Oikonomokis et al. [35]	156	Abdominal Oncologic	Recurrence rate	1.8	NS
			1 year survival	1.2	NS
			2 year survival	2.3	NS
Kusonoki et al. [36]	62	Abdominal Oncologic	Incidence of intestinal obstruction prior to ileostomy closure	2.2	0.60
			Incidence of intestinal obstruction following ileostomy closure	2.9	0.22
			Incidence of grade 0 adhesions midline	40.6	
			Incidence of grade 0 adhesions stoma	Cannot be calculated—0 events in control group	
			5 year survival	1.6	
Uchida et al. [32]	278	Abdominal Oncologic	No control group	n/a	n/a
Hayashi et al. [37]	144	Abdominal Oncologic	Incidence of small bowel obstruction	1.7	NS
Park et al. [38]	427	Abdominal	Incidence of early postoperative bowel obstruction	2.7	NS
		Oncologic	Incidence of readmission for early postoperative bowel obstruction	1.7	NS
Khaitan et al. [20]	19	Abdominal laparoscopy	No control group	n/a	n/a
Shinohara et al. [23]	8	Abdominal laparoscopy	Feasibility study	n/a	n/a
Ortiz and Awad [25]	n/a	Abdominal laparoscopy	Feasibility study	n/a	n/a
Kawamura et al. [29]	36	Abdominal laparoscopy	Ileostomy take down time	Cannot be calculated	0.023
			Seprafilm vs. no treatment	—no SD or SEM reported	
Kawamura et al. [6]	282	Abdominal laparoscopy	Incidence of 2-year adhesive postoperative bowel obstruction	7.8	0.021
Klinger et al. [45]	1	Abdominal	Case report	n/a	n/a
Trickett et al. [46]	4	Abdominal	Case report	n/a	n/a
Remzi et al. [47]	3	Abdominal	Case report	n/a	n/a
David et al. [48]	1	Abdominal	Case report	n/a	n/a
Tyler et al. [49]	3	Abdominal	Case report	n/a	n/a
Mizuno et al. [39]	9	Pediatrics abdominal	No control group	n/a	n/a
Ong et al. [40]	3	Pediatrics abdominal	No control group	n/a	n/a
Inoue et al. [41]	122	Pediatrics abdominal	Incidence of adhesions	6.7	0.007
			Incidence of greater grade 1 adhesions	12.8	0.0009
			Reoperative time	1.1	0.04
			Blood loss ≥ 3 g/kg	Cannot be calculated	0.09
			Blood loss < 3 g/kg	—no SD or SEM reported	
Winfield et al. [42]	18	Pediatric abdominal	No control group	n/a	n/a
Fushiki et al. [43]	52	Obstetrics	Incidence of adhesions	11.5	0.001
			Operative time to delivery	Cannot be calculated—only range reported	0.014
			Operative time total	Cannot be calculated—only range reported	0.009
			Blood loss	Cannot be calculated—only range reported	0.134
			Severity of adhesions	Cannot be calculated—only range reported	0.001
Diamond et al. [4]	127	Gynecologic	Mean number of sites adherent to the uterus	0.8	<0.0001
			Severity	0.5	<0.01
			Extent	0.4	<0.01
Tsuji et al. [44]	63	Gynecologic	Incidence of uterine adhesions compared o no treatment control	20	0.0003
			Incidence of uterine adhesions compared to Dextran 40	14.4	0.0004
			Incidence of uterine adhesions compared to Beriplast	18	0.0005
			Incidence of peritoneal adhesions compared to no treatment control	13.5	0.001
			Incidence of peritoneal adhesions compared to Dextran 40	2.5	0.2557
			Incidence of peritoneal adhesions compared to Beriplast	4.3	0.0818
			Incidence of adnexal adhesions compared to no treatment control	72	<0.0001
			Incidence of adnexal adhesions compared to Dextran 40	6.8	0.0098
			Incidence of adnexal adhesions compared to Beriplast	1.2	0.855
			AFS Score compared to no treatment control	1.5	<0.0001
			AFS Score compared to Dextran 40	0.9	0.0223
			AFS Score compared to Beriplast	0.3	0.8208
Takeuchi et al. [21]	114	Gynecologic laparoscopy	No control group	n/a	n/a
Chuang et al. [22]	127	Gynecologic laparoscopy	No control group	n/a	n/a
Fenton and Fanning [24]	15	Gynecologic laparoscopy	Feasibility study	n/a	n/a
Lipetskaia et al. [26]	171	Gynecologic laparoscopy	Feasibility study	n/a	n/a
Bristow et al. [33]	21	Gynecologic Oncologic	Mean pelvic adhesion score versus mean abdominal adhesion score (internal control)	1.9	0.002
			Mean pelvic adhesion score versus mean pelvic adhesion score (historical control)	1.7	0.004
Bristow et al. [50]	n/a	Gynecologic Oncologic	No control group—cost effectiveness	n/a	n/a
Tan et al. [34]	202	Gynecologic Oncologic	5 year disease free survival	0.6	NS
			5 year overall survival	0.6	
			30 day bowel obstruction	3.4	
Leitao et al. [17]	423	Gynecologic Oncologic	Incidence of intraabdominal collections	0.3	0.0009
Tabata et al. [18]	371	Gynecologic Oncologic	Incidence of early postoperative small bowel obstruction	5.0	<0.05
			Incidence of surgical site infection	2.0	NS

^a^For continuous outcomes, effect size is the (control group mean − the Seprafilm mean) divided by the pooled standard deviation for the 2 groups [ES = (M1 − M2)/pooled SD], and for binomial outcomes, effect size is the odds ratio or the ratio of the odds of a success for the Seprafilm group to the odds of a success for the control group [ES = (ad)/(bc)]

### Uses of Seprafilm following abdominopelvic surgery

The safety and efficacy of Seprafilm in abdominopelvic laparotomy were initially evaluated in two randomized controlled multicenter clinical trials. In one investigation, 183 patients with ulcerative colitis and familial polyposis undergoing colectomy with ileal pouch anal anastomosis and temporary loop ileostomy were enrolled [3]. The incidence, extent, and severity of adhesions to the underside of the abdominal wall incision were evaluated at the time of ileostomy closure. Absence of adhesions was seen in 51 % of Seprafilm treated patients, while only 6 % of control patients had no adhesions (*p* < 0.00000000001). Dense adhesions were observed in 58 % of control patients but in only 13 % of the Seprafilm treated patients (*p* < 0.0001). There was no statistically significant difference observed between the incidence of adverse events in the Seprafilm and control groups (*p* > 0.05) [3]. The most common side effects noted in the Seprafilm and control groups, respectively, were small bowel obstruction (9 % and 10 %), abscess (8 % and 2 %), nausea/vomiting/diarrhea (4 % and 5 %), pulmonary embolism (4 % and 0 %), deep venous thrombosis (2 % and 1 %), ileus (2 % and 1 %), fever (2 % and 0 %), and adrenal insufficiency (2 % and 0 %).

In a second multicenter, randomized trial, 127 women undergoing uterine myomectomy were included [4]. Patients were randomly assigned to receive the application or no application of Seprafilm to the anterior and posterior surfaces of the uterus following the myomectomy via laparotomy. Postoperative adhesion formation was evaluated during a second look laparoscopy performed an average of 23 days later. The mean number of abdominopelvic locations adherent to the uterus was significantly less in the Seprafilm group [4.98 (*n* = 49)] as compared with the control group [7.88 (*n* = 48)] (*p* < 0.0001). In addition, patients in the Seprafilm group had a significant reduction in the severity of adhesions (*p* < 0.01), the extent of adhesions (*p* < 0.01), and the area of uterus involved in the adhesions (*p* < 0.02). There was no statistically significant difference in the occurrence of adverse events between the treatment and control groups. Further, no adverse event was considered to be definitely related to Seprafilm.

Subsequently, the comparative efficacy of Seprafilm use in abdominal laparotomies was evaluated in a series of 30 patients undergoing colectomy [5]. The incidence of adhesions at Seprafilm treated sites was 36.7 % as compared to 63.3 % in the control sites within the same patient (*p* < 0.05). A comparison of this group of Seprafilm treated patients to a historical control that underwent abdominal laparotomy without Seprafilm showed that the incidence (*p* < 0.05) and severity (*p* < 0.01) of adhesions were lower in the Seprafilm group as compared to the historical controls.

In a series of individuals undergoing Billroth I anastomosis following distal gastrectomy, postoperative small bowel obstruction was examined in the absence (*n* = 169) or presence (*n* = 113) of Seprafilm use [6]. Adhesive related obstructions were significantly less in patients treated with Seprafilm, with rates of obstruction within the first 2 years of surgery being over sixfold higher (0.9 % with Seprafilm as opposed to 6.5 % without Seprafilm).

A large (*N* = 1,791), post-marketing, randomized, controlled-trial in patients undergoing laparotomies for small bowel obstruction, inflammatory bowel or diverticular disease was conducted to further evaluate the incidence of abscess, pulmonary embolism and foreign body reaction and the prevention of adhesive small bowel obstruction following Seprafilm use [7, 8]. Results from this trial demonstrate that compared to the no treatment control, Seprafilm treatment reduced the relative risk of a first adhesive small bowel obstruction by 47 % (1.8 % vs. 3.4 %, treatment vs. control, respectively, *p* = 0.044) in colorectal patients, where the outcome was verified by direct visualization [8]. Seprafilm treatment was found to have no effect on small bowel obstructions in which reoperation and direct visualization was not performed. There were no reports of foreign body reactions. Additionally, no relationship was observed between Seprafilm and the incidence of pulmonary embolus. However, a retrospective post hoc analysis suggested that wrapping Seprafilm around a newly created anastomosis was associated with a statistically significant increase in anastomotic leak related adverse events (fistula, leak, abscess, peritonitis, and sepsis). Based upon this data, the Seprafilm labeling has been updated to advise against wrapping Seprafilm directly around a newly created anastomotic suture or staple line. A multivariate analysis of factors that might affect the incidence of leak related events showed that wrapping of Seprafilm, lower body mass index, use of steroids, and preexisting abscess were predictors of leak related events. The increase in risk of leak related events with wrapping of Seprafilm [odds ratio, 2.7, 95 % confidence interval (1.8, 4.0)] was similar to the risk associated with the use of steroids [odds ratio, 1.9, 95 % confidence interval (1.3, 2.7)].

In another study, among 121 subjects undergoing intestinal resection, who received Seprafilm beneath the midline incision, 11 (9.1 %), developed postoperative ileus, with a mean time of onset of 16.1 ± 12.1 days [9]. In a subgroup who underwent a repeat surgical procedure, sites at which Seprafilm was placed beneath the anterior abdominal wall, had a lower incidence of adhesions (11/27, 40 %) as compared to untreated sites (24/27, 88.9 %, *p* = 0.0004).

A recent report purported to summarize the outcome of 4203 patients by way of a meta-analysis [10]. The authors concluded that Seprafilm use decreased intraabdominal adhesions after general surgical procedures, but did not reduce postoperative bowel obstruction, and was associated with increased intraabdominal abscess and bowel anastomotic leaks. Three letters to the Editor were subsequently published which criticized the methodology used for the meta-analysis. The deficits noted were that the authors included patients in the Seprafilm group that were treated with a different adhesion product, the 4,203 patients included represented an extremely diverse population of patients and surgical procedures, the data on effectiveness in reducing SBO included adverse event reporting and a definition of SBO which included paralytic ileus, and a failure to include published reports on the appropriate use of Seprafilm [11–13]. Two of the letters also reported potential errors in data abstraction from the initial publications [11, 13].

#### Small bowel obstruction

In a retrospective cohort study, the incidence of small bowel obstruction and enterolysis after Seprafilm use was compared to the incidence in a matched group of historical controls who did not receive Seprafilm [14]. A trend was noted in favor of the Seprafilm-treated patients regarding the incidence of small bowel obstruction (4.6 % in the Seprafilm group; 6.7 % in the control group, *p* = ns) and enterolysis for obstruction (1.5 % in the Seprafilm group; 3.9 % in the control group, *p* = ns).

In another study, Seprafilm was evaluated in 51 patients undergoing transabdominal aortic aneurysm surgery [15]. The authors reported that placement of Seprafilm over the anterior surface under the midline incision (*n* = 21) was associated with a significantly lower rate of postoperative small bowel obstruction, 0 %, when compared to a no treatment control group (*n* = 30), 20 % (*p* < 0.05).

In a retrospective study of 367 patients who had undergone elective gastrointestinal laparotomy, Seprafilm was reported to significantly lower the incidence of early postoperative bowel obstruction (EPSBO) when compared to matched controls [16]. One hundred eighty-four Seprafilm patients were compared with 183 no treatment patients. The incidence of EPSBO was 6.5 % in the treatment group versus 14.2 % in the control group. Surgical site infection rates were similar in both groups.

In a retrospective cohort study of 423 women with gynecologic malignancies undergoing a laparotomy (Seprafilm, *n* = 219; control, *n* = 204), there was no significant difference in the number of subjects with a small bowel obstruction or in the incidence of women who required a repeat operation for a small bowel obstruction [17]. However, there was a higher rate of intraabdominal fluid collection among Seprafilm treated subjects (4.1 %) as compared to controls (0.5 %, *p* < 0.02). In subgroup analysis, this difference was primarily attributable to women who underwent large bowel resection; there was no significant difference in the rates of intraabdominal fluid collection among women without large bowel resection.

In another report, a comparison of early postoperative small bowel obstruction in women who had placement of Seprafilm at the conclusion of surgery for gynecologic malignancies was compared with a historical control group [18]. Seprafilm use was associated with a significantly reduced rate of early small bowel obstruction as compared to the control group (6/191, 3.1 % vs. 25/180, 13.9 %, respectively, *p* < 0.05). Logistic regression analysis demonstrated Seprafilm use and conduct of pelvic lymphadenectomy as independent indicators of a reduction in early small bowel obstruction. These authors also reported that Seprafilm use was not associated with an increased risk of postoperative surgical infections (3.6 % vs. 6.7 % incidence, respectively).

#### Laparoscopic surgery

Although not approved in the USA for laparoscopic use, Seprafilm has been used laparoscopically in a case series of 19 patients suffering from chronic abdominal pain that underwent laparoscopic adhesiolysis with placement of Seprafilm [19, 20]. At an average follow-up of 9.6 months, 14 patients discontinued pain medication, 2 were taking NSAIDS, and 3 needed round the clock narcotics. One patient underwent a diagnostic laparoscopy 6 months after Seprafilm placement, and no adhesions were found.

Six additional publications have detailed methods of laparoscopic delivery of Seprafilm; the initial three studies delivered the intact Seprafilm barrier, while the three others described endoscopic delivery of a Seprafilm slurry. The first study published in 2006 describes the safe and efficient laparoscopic placement of the intact Seprafilm barrier in 114 patients undergoing myomectomy with a “purpose-built introducer” [21]. A second paper details laparoscopic Seprafilm placement in 127 gynecologic patients by way of rolling the membrane into a cylinder and subsequent placement through a 10 mm trocar sleeve [22]. A third publication describes a “flag” technique for Seprafilm placement in 8 patients undergoing laparoscopic abdominal surgery [23]. Other laparoscopic publications detail the creation of a slurry with the Seprafilm barrier. A Seprafilm slurry has been created by mixing 20 cc of sterile saline per 13 × 15 cm sheet of Seprafilm by one group, with back-loading into a leur lock syringe for delivery through a laparoscopic irrigator [24]. Fifteen patients undergoing gynecologic procedures were treated in this fashion with adherence noted to tissue including dependent surfaces and the anterior abdominal wall. The authors noted no adverse events which were related to Seprafilm or to laparoscopic application of the slurry. In similar reports, other authors [25, 26] described use of warm saline to create the slurry, with the Seprafilm solution placed in a Toumey syringe with a Robinson catheter, with the distal top cut off attached to the syringe, and then put through a laparoscopic trocar port for intraperitoneal delivery. A laparoscopic grasping instrument was utilized to direct or guide the catheter tip to the desired intraperitoneal sites for laparoscopic application [25, 26].

#### Closure of defunctioning loop ileostomy

In a prospective randomized study, application of Seprafilm around the limbs of a defunctioning loop ileostomy was found to reduce peristomal adhesions and to facilitate early stomal closure at 3 weeks with minimal complications [27]. A comparison of peristomal adhesions at closure between the Seprafilm and control groups at 3 weeks, showed that the Seprafilm group had a significant reduction in the overall mean adhesion scores as compared to the control group (5.81 ± 0.5 vs. 7.82 ± 0.6, respectively; *p* = 0.02 ). The number of patients with dense adhesions was also reduced in the Seprafilm group as compared to the control group. However, there was no statistically significant difference in the time taken, or difficulty encountered, during ileostomy closure in the groups. However, a trend for easier closure was observed in the Seprafilm group.

Another study assessed the incidence and severity of adhesions around a loop ileostomy and analyzed the length of time and morbidity associated with mobilization and closure of the ileostomy, with and without the use of Seprafilm [28]. A total of 191 patients with loop ileostomies were randomly assigned to either receive Seprafilm under the midline incision and around the stoma (Group I), only under the midline incision (Group II), or neither (Group III). At ileostomy closure, adhesions were graded and operative morbidity assessed. Significantly more untreated patients (Group III) had adhesions around the stoma than those who received Seprafilm treatment of the midline incision and stoma (Group I; 95.2 % vs. 82.3 %, *p* = 0.021). Mean operative times per treatment group were 27, 25, and 28 min, respectively (*p* = 0.38). There was no significant difference in the number of patients needing myotomy or enterotomy (29, 27 and 24 patients, respectively), nor in the number of postoperative complications (7, 9 and 7 patients, respectively).

In a recent report, “Sushi roll” wrapping of the ileostomy proximal and distal limbs and covering of the corresponding mesentery was performed in 18 subjects [29]. Comparing with a control group of 18 subjects, those in whom Seprafilm was utilized had a significant reduction in the time required for ileostomy closure at the subsequent procedure (107 vs. 121 min, respectively; *p* = 0.023).

#### Infection

In a published prospective, randomized clinical study evaluating the use of Seprafilm in the presence of peritonitis, Seprafilm reduced the severity but not the incidence of adhesions [30]. Abnormal postoperative wound healing was seen in 8/21 patients in the Seprafilm group and 3/21 patients in the control group. In the Seprafilm group, 4 patients had mild to moderate wound infection, 2 had abscesses related to the midline, and 2 had wound dehiscence. In the control group, three patients had abscesses. A publication on the long-term follow-up of 35 of these patients (16 Seprafilm and 19 control patients) reported that the incidence of chronic abdominal complaints (pain, nausea, obstipation) was significantly lower in Seprafilm patients when compared to control patients [31]. There was no difference in the incidence of small bowel obstruction between the groups (Seprafilm = 0 patient; control = 2 patients).

An additional study of Seprafilm in presence of infection was conducted on a cohort of 278 consecutive patients, who had undergone radical surgery for colorectal cancer with the placement of Seprafilm under the midline incision. There was no increase in the rate of septic conditions nor was there aggravation of postoperative inflammatory responses reported [32].

#### Malignancy

In a prospective clinical study, 14 women undergoing primary cytoreductive surgery with radical oophorectomy for locally advanced epithelial ovarian cancer received Seprafilm in the pelvic cavity [33]. The abdominal wall incision which did not receive Seprafilm served as control. A statistically significant decrease in mean adhesion scores and the areas involved with adhesions was noted in Seprafilm-treated sites when compared to control sites. There were no instances of anastomotic leak and no perioperative complications attributable to the treatment with Seprafilm. Only one patient had positive biopsy results indicating persistence of cancer.

Tan et al. examined whether Seprafilm altered cancer survival in women undergoing surgery for primary peritoneal, ovarian, and fallopian tube malignancies [34]. They suggest a need for such a study was based on reports that hyaluronan may promote tumor growth. Among 202 women, prospectively followed with a median follow-up of 2.1 years, 139 achieved a disease free interval. The authors reported that in comparing those who received Seprafilm (*n* = 88) versus those that did not (*n* = 122), there was no difference in overall survival, disease free survival, or the rate of immediate postoperative complications.

A retrospective chart review of 156 patients who had curative surgery for non-metastatic colorectal cancers, found no difference in 1- and 2-year disease-free survival among the patients who were administered Seprafilm (88 % and 85 %, respectively) when compared to patients who did not receive any Seprafilm (85 % and 72 %), *p* = 0.44 [35]. After an average follow up of 11.4 months, there was no significant difference between the incidence of peritoneal carcinomatosis, recurrence of tumor, or elevation of carcinoembryonic antigen between the two groups.

Seprafilm was reported to reduce adhesions to the midline and stoma and have no adverse effect on oncologic outcome in patients, treated with chemo and radiation therapy, following radical resection of rectal carcinoma [36]. Thirty-two patients were treated with Seprafilm and 30 received no treatment following their colon resection. Seprafilm significantly reduced the severity and extent of adhesions to the stoma and midline incision which was associated with reduced surgical time, blood loss and the extent of the incision required at ileostomy closure. Seprafilm was not associated with any postoperative complications and did not affect recurrence or survival rates.

A randomized trial was conducted in 144 patients who underwent gastrectomy in whom two sheets of Seprafilm were placed between the abdominal wall and the small bowel (*n* = 70) or were not (*n* = 74) [37]. Seprafilm use was associated with a non-significant reduction in the overall incidence of small bowel obstruction (5.7 % versus 9.5 % in the control group), as well as a non-significant reduction in the cumulative incidence of obstruction of the small bowel (6.2 % vs. 12.2 % in the control group at 36 months). There was no difference in the rates of postoperative complications (32.7 % vs. 39.7 %, respectively) nor was there an identified effect on intestinal, liver, or kidney function.

In another recent study to examine reduction of adhesive bowel obstruction following surgery for colorectal cancer, 427 subjects were randomized to receive (*n* = 185) or not to receive (*n* = 242) one piece of Seprafilm over the denuded pelvic inlet [38]. Bowel obstruction was defined as the presence of nausea, vomiting, and abdominal distention, in combination with a radiologic obstructive bowel pattern. There was no difference in the clinicopathologic parameters or non-obstructive complications between the two groups. However, the incidence of early postoperative small bowel obstruction was less in the Seprafilm treated subjects than in the controls (2.7 % vs. 7.0 %, respectively, *p* = 0.045). A similar trend which did not reach significance was identified in the later postoperative follow-up period (2 % vs. 4.6 %, respectively).

#### Pediatric laparotomies

In a case series of nine pediatric patients (age range, 25 days to 8 years; mean age, 1.7 years), Seprafilm was used in 10 laparotomies [39]. No significant difference was observed in the hematological parameters and renal and hepatic function tests. Only one patient developed an adverse event (ileus) that was managed conservatively. One patient who underwent a second laparotomy had no adhesion at the site of placement of Seprafilm. Another case series of 4 children (ages 57 days to 8 years) reported on Seprafilm use in pediatric patients for a variety of hepatobiliary conditions [40]. No adverse events were noted in any of these patients. One patient (age 57 days) who underwent a second laparotomy had no adhesion in the area where Seprafilm was applied.

In a randomized cohort study in 122 pediatric patients Seprafilm was reported to safely and effectively reduce the incidence and severity of adhesions under the midline incision [41]. Patients were randomized, following laparotomy, to either receive Seprafilm placement under the midline incision (*n* = 67) or no treatment (*n* = 55). In the Seprafilm group, 18 patients required a planned second laparotomy and 4 of the 18 required a third procedure. Seprafilm was applied during each repeat procedure. In the control group, 13 of 55 patients required a planned second laparotomy and 4 of these patients required a third. Efficacy comparisons were conducted on the 18 Seprafilm patients versus the 13 control. At second surgery, 59.1 % of Seprafilm patients had no adhesions versus 17.6 % in the control group. Severity scores were also lower in the Seprafilm treated patients versus the control group. Seprafilm patients’ severity scores did not change between the second and third surgeries. In a retrospective report of 18 pediatric laparotomy patients (25–18 years), no patients experienced small bowel obstruction, intraperitoneal abscess or a localized inflammatory reaction following Seprafilm use. Seprafilm was reported to be successfully used without complications [42].

#### Obstetrics and gynecologic procedures

##### Cesarean section

Use of Seprafilm for preventing adhesions during cesarean section was evaluated in women undergoing repeat cesarean section who had received Seprafilm during the previous cesarean section (*n* = 6) and compared to women who had not (*n* = 22) [43]. None of the patients who had received Seprafilm showed any adhesions at the time of repeat cesarean section while 54.5 % of the control women did. The duration of surgical procedure and the amount of blood loss did not differ between the two groups; however, a trend was noted in favor of Seprafilm for the duration of time between the start of procedure and the delivery of the baby [an average 4.5 min (range, 3–6 min) in the Seprafilm group and 6.4 min in the control group (range: 2–10 min)] *p* = 0.057.

##### Myomectomy

In 63 gynecological patients undergoing myomectomy, Seprafilm was reported to have the lowest incidence of uterine and peritoneal adhesion formation and AFS scores when compared to all anti-adhesion materials tested [44]. Seprafilm treated patients (*n* = 21) were compared to Dextran 40 (*n* = 17) patients, factor 13 with fibrinogen (Beriplast) treated patients (*n* = 12), and no treatment control (*n* = 13). Uterine adhesions were present in 14 % of the Seprafilm patients, 71 % of the Dextran 40 patients, 75 % of the Beriplast patients and 77 % of the no treatment control patients (*p* ≤ 0.0005). Seprafilm was reported to be more effective in preventing serosal uterine adhesions following myomectomy than all the other interventions tested.

#### Possible adverse effects

Published case reports have described the appearance of a foreign body reaction coincident with the use of Seprafilm. The first case was a 69-year-old man who underwent laparotomy with the placement of Seprafilm and became febrile 12 days after surgery [45]. An exploratory laparotomy revealed an intense inflammatory reaction under the midline incision, which on biopsy contained foreign body granulomas. The patient received steroids and antibiotics after surgery and made a full recovery. The second case was a 71-year-old woman with 4 previous abdominal surgeries, who underwent laparotomy with Seprafilm placement [46]. The patient developed obstructive symptoms and a second laparotomy on the 21st postsurgical day revealed a dense, thick, glue-like mass involving the small intestine and transverse colon. The biopsy revealed foreign body granulomas. The patient underwent colectomy and anastomosis. An iatrogenic tear in the transverse colon led to feculent peritonitis resulting in death.

In a case series of three patients, all developed abdominal fluid collection with fever, signs of peritonitis, and raised neutrophil counts within 4–7 days after receiving Seprafilm during laparotomy [47]. All three underwent laparotomy and a cloudy ascitic fluid was seen in each case. Copious washing of the abdominal fluid in each case was followed by improvement in the patient’s condition.

A paradoxical inflammatory reaction to Seprafilm was attributed to extensive adhesion formation early in the postoperative period in a 70-year-old woman who had undergone a low anterior resection [48]. Exploratory surgery on postoperative day 11 revealed extensive dense adhesions involving the abdominal wall, omentum, and small bowel. Pathology revealed diffuse inflammation with multiple giant cells suggesting a foreign body reaction. The authors conclude that reports of this kind are uncommon.

Collections of sterile intra-abdominal fluids were identified in three subjects following the use of Seprafilm in colorectal surgery [49]. The subjects of each received 2 or 3 sheets of Seprafilm. Two to 3 weeks later, they were thought to have a possible abscess or had a slow postoperative course. A CT scan identified the fluid which was later determined to be a sterile collection.

#### Cost-effectiveness

Notwithstanding the repeated reports of clinical efficacy of Seprafilm described previously in the review, many hospital and health care administrators have remained unconvinced of the value of routine use of anti-adhesion adjuvants. Bristow and colleagues addressed this issue in a cost-effectiveness analysis of women with stage 1B cervical cancer undergoing a radical hysterectomy with pelvic lymphadenectomy, in the presence or absence of Seprafilm [50]. The authors utilized a decision analysis model, incorporating factors identified by the Panel on Cost-Effectiveness in Health and Medicine, which was convened by the US Public Health Service. Clinical assumptions were based on Phase III trials where available, and otherwise on Phase II trials, retrospective case series and case reports. Use of Seprafilm in these models was efficacious both in term of cost to society (incremental cost of non-use $1,112 per patient, with greater quality adjusted life years), as well as cost to third party payers (incremental cost of non-use $383 per patient, with greater quality adjusted life years). The authors conclude that “an adhesion prevention strategy utilizing Seprafilm in patients undergoing radical hysterectomy and pelvic lymphadenectomy for Stage 1B cervical cancer, as well as other similar procedures, is cost effective from both the perspective of society as a whole and that of a third party payer.” Furthermore, they conclude that “With current concerns over the escalating economic burden of healthcare, straightforward clinical intervention such as the prevention of adhesion related morbidity, offers a real opportunity for significant cost savings in health care expenditure.”

## Summary

The safety and efficacy of Seprafilm use has been demonstrated in abdominopelvic surgery. Retrospective analysis from the result of a randomized controlled trial suggests that wrapping Seprafilm around a newly created anastomosis may be associated with an increase in anastomotic leak related adverse events (fistula, leak, abscess, peritonitis, and sepsis). Based upon this data, Seprafilm labeling has been updated to warn against wrapping Seprafilm directly around a newly created anastomotic suture or staple line. Additional studies have been undertaken in abdominopelvic procedures to explore the use of Seprafilm in the presence of overt infections, malignancy, in pediatric patents, in early ileostomy closure, laparoscopic surgery, and cesarean sections. While the results of these studies are interesting, appropriately designed randomized controlled clinical trials still need to be conducted. Additionally, the results of one cost-effectiveness study are encouraging but more investigation is warranted. Also, other future studies are needed to further delineate the molecular biologic processes leading to normal peritoneal healing as opposed to adhesion development, which would allow targeted interventions to improve adjuvant efficacy. Such approaches could utilize anti-adhesion products to physically separate otherwise opposing tissue surfaces while also serving as a carrier for the delivery of drugs and/or biologics to further improve the efficacy of anti-adhesion adjuvants.
